# The Association Between Promoter Tandem Repeat Polymorphism (pVNTR) and CYP2C9 Gene Expression in Human Liver Samples

**DOI:** 10.3390/genes16020213

**Published:** 2025-02-11

**Authors:** Abelardo D. Montalvo, Yan Gong, Joseph M. Collins, Danxin Wang

**Affiliations:** Department of Pharmacotherapy and Translational Research, College of Pharmacy, Center for Pharmacogenomics and Precision Medicine, University of Florida, Gainesville, FL 32610, USA; admontalvo@ufl.edu (A.D.M.); gong@cop.ufl.edu (Y.G.); jcoll86@cop.ufl.edu (J.M.C.)

**Keywords:** cytochrome P450, CYP2C9, promoter, tandem repeat polymorphism, gene expression

## Abstract

CYP2C9 metabolizes approximately 20% of clinically administered drugs. Several single-nucleotide polymorphisms (SNPs) of CYP2C9 (e.g., *2, *3, *8, and rs12777823) are used as biomarkers to predict CYP2C9 activity. However, a large proportion of variability in CYP2C9 expression remains unexplained. Background/Objectives: We previously identified a variable number tandem repeat (pVNTR) polymorphism in the CYP2C9 promoter. The short repeat (pVNTR-S) showed reduced transcriptional activity in reporter gene assays and was associated with decreased CYP2C9 mRNA expression. However, because pVNTR-S is in high linkage disequilibrium (LD) with CYP2C9*3 in the European population, whether pVNTR-S directly impacts CYP2C9 expression remains unclear. The objective of this study was to clarify the association between the pVNTR-S and CYP2C9 mRNA expression in human liver samples and to assess its impact on CYP2C9 expression independently of known CYP2C9 biomarkers. Methods: Gene expression was measured by real-time qPCR. SNPs and pVNTRs were genotyped using SNapShot assays and fragment analysis, respectively. Associations between CYP2C9 and the pVNTR-S or SNPs were analyzed using multiple linear regression. Results: Our results showed that pVNTR-S was associated with lower CYP2C9 expression (34% reduction, *p*-value = 0.032) in human liver samples (*n* = 247), while the known CYP2C9 biomarkers (CYP2C9*2, *3, *8, or rs12777823) were not. These results suggest that pVNTR-S reduces CYP2C9 expression independently of known biomarkers. Therefore, pVNTR-S may explain additional variability in CYP2C9 expression when present alone or in conjunction with other CYP2C9 alleles.

## 1. Introduction

The cytochrome P450 family 2 subfamily C member 9 (CYP2C9) is responsible for the metabolism of ~20% of clinically relevant drugs. The CYP2C9 gene is located on chromosome 10q24 within a multigene cluster containing four CYP2C genes (CYP2C19, CYP2C9, CYP2C18, and CYP2C8) [1]. Genetic variants of CYP2C9 cause variable CYP2C9 enzymatic activities, affecting the treatment outcomes of many CYP2C9 substrate drugs, for example, coumarin anticoagulants (warfarin and acenocoumarol), antiepileptics (phenytoin), nonsteroidal anti-inflammatory drugs (ibuprofen, celecoxib, and meloxicam), and others [2]. Several single-nucleotide polymorphisms (SNPs) of CYP2C9, (e.g., CYP2C9*2, *3, and *8) are recommended by the Clinical Pharmacogenomics Implementation Consortium (CPIC) to be used to guide the therapy of many CYP2C9 substrate drugs [3] (also see Pharmgkb.org). However, large inter-person variability in CYP2C9 metabolism [4] remains not fully unaccounted for, particularly in individuals of non-European descent, resulting in unpredictable drug clearance and adverse drug reactions [3].

While coding SNPs in CYP2C9 have been extensively studied, polymorphisms that affect CYP2C9 expression remain uncharacterized, likely contributing to variation in CYP2C9 activity. For example, rs12777823 is located ~300 kb away from CYP2C9 and is associated with a reduced stable warfarin dose requirement [5]; the underlying mechanisms of how rs12777823 affects CYP2C9 expression/activity remain unclear. In addition to *cis*-acting genetic variants, *trans*-acting factors like transcription factors (TFs) also affect CYP2C9 expression. These include the pregnane X receptor (PXR or NR1I2), constitutive androstane receptor (CAR or NR1I3), peroxisome proliferator-activated receptor alpha (PPARA), aryl hydrocarbon receptor nuclear translocator (ARNT), hepatic nuclear factor 4 (HNF4A), forkhead box protein A2 (FOXA2), and estrogen receptor alpha (ESR1) [4,6,7].

A promoter variable number tandem repeat (pVNTR) polymorphism was previously identified 4 kb upstream of the CYP2C9 translation start site (NC_000010.11; 94934570–94934705; GRCh38) [8]. There are three pVNTR alleles of different lengths: short (pVNTR-S; 417–438 bp), medium (pVNTR-M; 446–488 bp), and long (pVNTR-L; 512–522 bp). The pVNTR-S is associated with allelic mRNA expression of CYP2C9 in human liver samples and reduced transcriptional activity in reporter gene assays [8]. However, pVNTR-S is in strong linkage disequilibrium (LD) with CYP2C9*3 in the European population, so the independent effects of pVNTR-S on CYP2C9 expression and activity could not be assessed in this population [8]. Several studies found varied allele frequencies and LD patterns of pVNTR-S in European, African, Egyptian, Spanish, and Jordanian populations [8,9,10]. For example, compared to European Americans (EAs), the allele frequency of the pVNTR-S is higher in Egyptians, and the LD between pVNTR-S and CYP2C9*3 is lower in Africans [8,9]. Therefore, in non-European populations, it may be possible to test the independent effects of pVNTR-S on CYP2C9 expression, which has not been conducted in previous studies. This study aimed to investigate whether pVNTR-S is independently associated with CYP2C9 expression in human liver samples derived from African American (AA) and EA donors by genotyping and adjusting for other SNPs that are in LD with it.

## 2. Materials and Methods

### 2.1. Human Liver Samples

Human liver biopsy samples derived from AA (*n* = 134) and EA donors (*n* = 113) were obtained from the Cooperative Human Tissue Network (CHTN, chtn.cancer.gov). All samples were biopsies of histopathologically confirmed normal liver tissues from cancer patients (most of them with colon cancer metastasized to the liver). Only information on donor’s age, sex, and race was available. All available samples at the time of procurement were included. The University of Florida Institutional Review Board approved the human tissue study.

### 2.2. DNA and RNA Extraction

DNA and RNA extraction was performed as described previously [7]. Briefly, DNA was extracted from the human liver samples using a Qiagen DNeasy kit per the manufacturer’s protocol (Qiagen, Germantown, MD, USA). RNA was extracted using the Zymo Research direct-zol RNA miniprep kit (Zymo Research, Irvine, CA, USA) according to the manufacturer’s protocol. Complimentary DNA (cDNA) was prepared using qScript Ultra Flex kit (Quantabio, Beverly, MA, USA) with gene-specific primers and oligo(dT), as described previously [7].

### 2.3. Genotyping

The CYP2C9 pVNTR polymorphism was genotyped using PCR with a fluorescently labeled primer followed by capillary electrophoresis, as described previously [8]. Briefly, CYP2C9 promoter DNA fragments containing pVNTR (417 bp–522 bp) were PCR amplified (primers and PCR conditions shown in Table 1). Then, one µL of diluted PCR products (a 1 to 5 dilution) was denatured and analyzed by SeqStudio (Thermofisher, Waltham, MA, USA) with GeneScan™ 600 LIZ^®^ (Thermofisher) as the size standard, according to the manufacturer’s protocol. The three pVNTRs of different lengths were identified based on previous studies [8,9,10]. Three SNPs, CYP2C9*2 (rs1799853), CYP2C9*3 (rs1057910), and CYP2C9*8 (rs7900194) were genotyped using multiplex SNapShot assays, as previously described [8]. Briefly, DNA fragments surrounding each SNP were amplified using primers and PCR reagents, as shown in Table 1. Then, 2 µL of the PCR products from each of the three PCR reactions were combined and treated with Exo I to remove the residual primers. This was followed by multiplex SNapShot assays as described in [11] with SNapShot reagent (Thermofisher, Waltham, MA, USA) and the primers listed in Table 1. The SNapShot products were then denatured and analyzed with SeqStudio using the GeneScan™ 120 LIZ^®^ size standard according to the manufacturer’s protocol. The genotyping data were analyzed using GeneMapper Software 5 (Thermofisher, Waltham, MA, USA). The rs12777823 genotype was extracted from whole genome genotyping results generated by the Genotyping Core Lab (Center for Genome Technology, University of Miami) using the Illumina GSAv3MD array (unpublished).

### 2.4. Gene Expression Quantification

Gene expression was quantified by real-time qRT-PCR with SYBR green and gene-specific primers using the Quantabio Q real-time PCR instrument (Quantabio, Beverly, MA, USA) as previously described [7]. The expression of β-actin served as an internal control. The relative expression of each gene was calculated using the following formula: the expression level of the tested gene = antilog2 (mean Ct value of β-actin—mean Ct value of the tested gene) × 10^6^. After log10 transformation, the expression level of all genes followed a normal distribution.

### 2.5. Statistical Analysis

Multiple linear regression models were used to test the association between pVNTR or other SNPs (CYP2C9*2, CYP2C9*3, CYP2C9*8, or rs12777823) and CYP2C9 gene expression. An additive model (coded as 0, 1, and 2 for zero, one, or two variant alleles, respectively) was used to test the association of each genotype, adjusting for age, race, and sex. Because previous reporter gene assays showed no differences in reporter activities between pVNTR-M and pVNTR-L [8], we combined pVNTR-M and pVNTR-L into one group, which represents the reference alleles (coded as 0), while assigning pVNTR-S as the variant allele (coded as 1 or 2 depending on the copy number). Several TFs (RXRA, NR1I2, NR1I3, ESR1, PPARA, HNF4A, FOXA2, AHR, and ARNT) known to be associated with CYP2C9 gene expression [6,7,12] were also included in the analysis. We used a backward stepwise linear regression approach to select the best set of covariates with a cut-off setting to stay in the model at a *p*-value < 0.05. The covariates included in the final model were age, race, sex, NR1I3, PPARA, ESR1, FOXA2, AHR, and ARNT. The assumptions of linear regression, constant variance (assumption of homoscedasticity), normally distributed residuals, linearity, and lack of multicollinearity between predictors were tested. The interactions among all TFs and the pVNTR were assessed to determine if one predictor’s effect is dependent on another’s presence. Statistical analyses were performed using R version 4.1.2. LD statistics between the SNPs and pVNTRs were obtained using the SNPstat software version 0.96 (https://www.snpstats.net/; accessed on 10 December 2024). Power calculation was performed using the R pwr package using the proportion of variance (R^2^) which was then used to calculate the effect size ƒ2 (https://cran.r-project.org/web/packages/pwr/, accessed on 26 January 2025). For the additively coded SNPs or pVNTR-S, the proportion of variance R^2^ was calculated using the equation R^2^ = 2p (1 − p)β^2^ where p is the allele frequency, and β is the linear effect size per allele calculated from the estimate (10^(estimate)) obtained from the multiple linear regression models. ƒ2 was then calculated using the equation ƒ2 = R^2^/(1 − R^2^). The alpha level was set at 0.05, and power was calculated using the pwr.f2.test function from the pwr package to compute the power for each linear regression model in the combined, as well as the separate AA and EA groups.

## 3. Results

### 3.1. Demographics Information of Liver Donors

Among the 287 patient samples, 247 were included in this study that had complete genotype and gene expression data. Demographic information of the 247 donors is shown in Table 2. The median age of the donors was 59 years and 51% were females. The average age of the EA donors was older than the AA donors (*p* = 0.036, Student’s *t*-test). There was no difference in the proportion of females between the AA and EA donors (*p* > 0.05, chi-squared test).

### 3.2. The Genotype and Allele Frequencies of pVNTR, CYP2C9*2, CYP2C9*3, CYP2C9*8, and rs12777823

**Figure 1:**
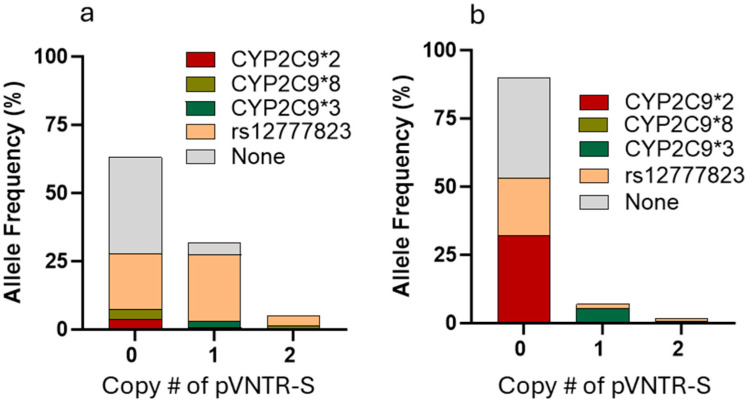
The distribution of CYP2C9*2, *3, *8, and rs12777823 in individuals with 0, 1, or 2 copies of pVNTR-S in AAs (**a**) or EAs (**b**).

Table 3 shows the genotype and allele frequencies for the polymorphisms tested in this study. The allele frequency of the pVNTR-S was higher in AAs than in EAs, while the frequency of pVNTR-L was lower. As expected, the minor allele frequency (MAF) of CYP2C9*2 and *3 was lower in AAs than in EAs, while the MAF of rs12777823 was higher in AAs than in EAs. CYP2C9*8 was common in AAs but absent in EAs. Table 4 shows the pairwise LD statistics between pVNTR-S and CYP2C9 *2, *3, *8, or rs12777823 in the AA and EA samples. pVNTR-S was in high LD with *3 in both AAs and EAs, with D’ being nearly 1. However, there was low r^2^ (r^2^ = 0.05) in AAs due to the lower allele frequency of CYP2C9*3 in this ancestry. As a result, there were numerous pVNTR-S samples that did not carry CYP2C9*3 (Figure 1). pVNTR-S was in partial LD with rs12777823 in AAs and EAs. Other pairs, like CYP2C9*2/pVNTR-S, CYP2C9*2/CYP2C9*3, and CYP2C9*3/CYP2C9*8, were in opposing LD and are unlikely to co-exist on the same chromosome, consistent with what is reported in the NCBI database [13].

### 3.3. The Association Between pVNTR-S and CYP2C9 Gene Expression

pVNTR-S was significantly associated with CYP2C9 expression after adjusting for age, sex, and race, with each pVNTR-S allele being associated with a 34% reduction in CYP2C9 expression (as compared to pVNTR-L and pVNTR-M combined) (*p*-value = 0.032) (Table 5). The significant association between pVNTR-S and CYP2C9 expression remained or improved after adjusting for TF expression levels (NR1I3, PPARA, ESR1, FOXA2, AHR, and ARNT) (*p* = 0.0007). Race was also a significant variable, with AA samples having 44% lower CYP2C9 expression than the EA samples (*p* = 0.0021), consistent with a previous report [7], while sex and age did not show significant associations (*p* > 0.05). pVNTR-S and race explained 6.7% of total variability, and pVNTR-S alone explained ~2%.

When the AA and EA data were analyzed separately, the association between the pVNTR-S and CYP2C9 expression was only observed in AAs (Table 5), possibly caused by the low pVNTR-S frequency in EAs. The other SNPs, CYP2C9*2, *3, *8, and rs12777823, were not associated with CYP2C9 expression (Table 5). We conducted a post hoc power calculation using the results from the multiple linear regression models to confirm that our study was sufficiently powered to detect an effect of the pVNTR-S on CYP2C9 expression. In the combined cohort, the power to detect the genotype effects for pVNTR-S, CYP2C9*2, and rs12777823 was sufficient (>95%), while the power for CYP2C9*3 and CYP2C9*8 was low (<60%). In the separate cohorts, the power for pVNTR-S was sufficient in AAs (94%) but low in EAs (65%). The power for rs12777823 was also sufficient in both the AA and EA cohorts (>95%) and for CYP2C9*2 in EAs, while the power was low (<80%) for CYP2C9*3 and CYP2C9*8 in both the AA and EA cohorts.

**Figure 2:**
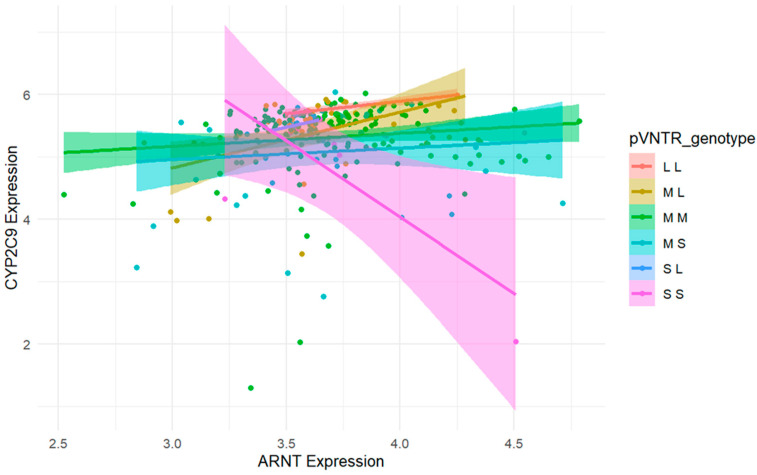
Interaction between pVNTR-S and ARNT. Intersecting lines indicate an interaction, while parallel lines do not. The interaction was only significant in homozygous pVNTR-S samples.

To evaluate whether the association between CYP2C9 and pVNTR-S depends on the expression of TFs, we tested the interactions among each TF and pVNTR-S in the model. There was a statistically significant interaction between ARNT and pVNTR-S (interaction *p* = 0.007). CYP2C9 expression was negatively correlated with ARNT expression only in samples with homozygous pVNTR-S. However, there was no correlation between CYP2C9 expression and ARNT expression in the samples with 1 or 0 copies of pVNTR-S (Figure 2). When testing the interactions in the AA and EA cohorts separately, no statistically significant interactions were observed for all TFs except between ARNT and pVNTR-S. The statistically significant interaction between ARNT and pVNTR-S remained in AAs (*p* value of interaction = 0.002) but was not observed in EAs (*p* value of interaction = 0.355) (Supplemental Figure S1).

## 4. Discussion

In this study, we tested the association between pVNTR-S and CYP2C9 expression in liver samples derived from AA and EA ancestries. We found a significant association between CYP2C9 expression and pVNTR-S, but not with the other known CYP2C9 SNPs (CYP2C9*2, CYP2C9*3, CYP2C9*8, and rs12777823) that are associated with decreased CYP2C9 activity [14,15,16] and that are currently used as biomarkers to predict CYP2C9 activity. This indicates that the association between pVNTR-S and CYP2C9 expression was not driven by other known SNPs in high LD with pVNTR-S. This result agrees with pVNTR-S causing reduced transcriptional activity in reporter gene assays [8] and suggests that pVNTR-S may contribute to additional variability in CYP2C9 expression.

The frequency of pVNTR-S in EAs was similar to previous reports in Caucasians [8], while the frequency of pVNTR-S in AAs was higher than in EAs (Table 3). The allele frequency of pVNTR-S in AAs was similar to those reported in Jordanians [10] but higher than previously reported in Blacks [8], possibly caused by heterogeneity within populations or by the different allele classification methods used. Therefore, there are large differences in pVNTR-S frequencies between different populations. We found that the length of pVNTR-S in AAs was ~2 bp longer (440 bp) than previously reported in EAs (438 bp) [8]. This indicates that the previous usage of a 417 bp–438 bp cut-off for pVNTR-S assignment may have misclassified pVNTR-S carriers in AA individuals as pVNTR-M, thereby underestimating the allele frequency of pVNTR-S in that study [8]. The two classification methods had no differences in pVNTR-S assignment in EAs.

None of the three coding SNPs tested (CYP2C9*2, *3, and *8) showed a significant association with CYP2C9 expression. This result was expected, as non-synonymous SNPs change protein structure rather than affecting gene expression [14,15,16]. We also did not find an association between the distal non-coding SNP rs12777823 and CYP2C9 expression. This was not expected, as the effect of rs12777823 is anticipated to reduce CYP2C9 expression because it is a distal non-coding SNP [5], and our sample size was sufficiently powered (>95%) to detect its effect. However, the mechanism underlying the association between rs12777823, CYP2C9 expression, and stable warfarin dose requirements remains unclear, and to our knowledge, this is the first report testing the association between rs12777823 and CYP2C9 expression.

pVNTR-S was previously reported to be associated with reduced stable warfarin dose in EAs, but the in vivo effects of pVNTR-S on CYP2C9 metabolism could not be separated from CYP2C9*3 effects due to high LD in EAs [8]. In contrast, although pVNTR-S is also linked to CYP2C9*3 in AAs, the frequency of pVNTR-S is higher, and the frequency of CYP2C9*3 is lower than in EAs, resulting in the frequent occurrence of pVNTR-S without CYP2C9*3 in AA individuals (Figure 1). Our results showed a significant association between pVNTR-S, but not CYP2C9*3, and CYP2C9 expression, which strongly indicates that pVNTR-S has an independent effect on CYP2C9 expression. Thus, when pVNTR-S co-exists with CYP2C9*3, pVNTR-S may further contribute to the reduced activity of CYP2C9, but it cannot serve as a separate biomarker for CYP2C9 activity due to its high LD with CYP2C9*3. However, when pVNTR-S does not co-exist with CYP2C9*3, as in individuals with African ancestry, pVNTR-S may serve as an additional biomarker to predict reduced CYP2C9 activity. Thus, pVNTR-S may improve CYP2C9 test panels for personalized drug therapy in certain populations where the LD between CYP2C9*3 and pVNTR-S is low.

The limitation of our study is that we tested the association between pVNTR-S and expression of CYP2C9 at the transcriptional level. It is unclear whether pVNTR-S is associated with CYP2C9 protein expression or activity. Moreover, although the liver samples used in this study are normal tissues, they were derived from individuals with cancers, and it is unclear whether the cancer environment affects the expression and regulation of CYP2C9. Therefore, whether pVNTR-S can serve as a biomarker warrants further investigation.

The mechanism underlying how pVNTR-S alters CYP2C9 gene expression remains unclear. Tandem repeats are abundant in human promoters [17] and are known to alter gene expression and DNA methylation patterns [18] and to modify disease susceptibility [19]. Our results showed an interaction between the pVNTR-S and the TF ARNT, with higher expression of ARNT being associated with decreased pVNTR-S effects (Figure 2). ARNT, also known as hypoxia-inducible factor 1β (HIF1β), is a ubiquitously expressed nuclear protein and a heterodimerization partner of another key CYP-gene regulator AHR [20]. ARNT regulates a wide range of cellular processes [21,22], and its expression is influenced by many conditions, like hypoxia and chronic inflammation [23,24]. Thus, factors that affect the expression of ARNT may modify the association between pVNTR-S and CYP2C9. However, the interaction between pVNTR-S and ARNT may be weak as it only occurs in homozygous pVNTR-S samples, and thus, the in vivo effect of this interaction requires further investigation.

## 5. Conclusions

We tested and found a significant association between a promoter tandem repeat polymorphism pVNTR-S and CYP2C9 expression in liver samples derived from AA and EA donors. None of the other common reduced-activity SNPs (i.e., CYP2C9*2, *3, *8, and rs12777823) in high LD with pVNTR-S showed significant associations with CYP2C9 expression. These results indicate that the association between pVNTR-S and CYP2C9 expression is independent of these other reduced-activity SNPs and that pVNTR-S may explain additional variability in CYP2C9 mRNA expression. We also found a potential interaction between pVNTR-S and the expression of the TF ARNT, a known CYP2C9 regulator. Larger scale studies are needed in different populations to further evaluate the in vivo effect of the pVNTR-S.

## Figures and Tables

**Table 1:** Sequence of primers.

PCR Primers	Primer Sequence	PCR Conditions
pVNTR	F: FAM-AGGGAACCAGAGAAGAAGGACA R: CCATCTCTGTCTTTTCATCTCATTC	JumpStart 2X PCR Mix. Denature at 95 °C for 3 min, then 40 cycles of [95 °C × 15 s 60 °C × 30 s, 72 °C × 1 min], followed by 72 °C × 10 min.
CYP2C9*2 & *8	F: GGTGCTGCATGGATATGAAGCA R: TCAAACCCCCGCTTCACAT	ToughMix 2X PCR Mix. 40 cycles of [98 °C × 10 s, 60 °C × 5 s, 68 °C × 1 s], followed by 68 °C for 3 min.
CYP2C9*3	F: ACTTACCCATGCCCCTTTGT R: ACCCGGTGATGGTAGAGGTT	JumpStart 2X PCR Mix. Denatured at 95 °C for 3 min, then 40 cycles of [95 °C × 15 s, 60 °C × 30 s, 72 °C × 1 min], followed by 72 °C for 10 min.
SNapShot primers	Primer sequence	SNapShot assay condition
CYP2C9*2 ^a^	GGGAAGAGGAGCATTGAGGAC	SNapShot reagent. 30 cycles of [95 °C × 10 s, 55 °C × 5 s, 60 °C × 30 s].
CYP2C9*3 ^a^	GGTGCACGAGGTCCAGAGATAC	
CYP2C9*8	TCTCAACTCCTCCACAAGGCAG	

^a^ Different numbers of Ts were added to the 5′ ends of these primers to enable multiplexing in genotyping.

**Table 2:** Demographics of liver donors.

	AA and EA	AA	EA	AA vs. EA, *p*-Value
Number (*n*)	247	134	113	
Age, years, median (range)	59 (0–97)	57 (0–97)	63 (14–83)	0.0366 ^a^
Female, *n* (%)	125 (51)	64 (48)	61 (54)	0.329 ^b^

^a^ Two-tailed Student’s *t*-test; ^b^ chi-squared test.

**Table 3:** The frequencies of tested alleles in AAs, EAs, and the combined cohort.

	AA and EA, *n* = 247	AA, *n* = 134	EA, *n* = 113
pVNTR			
Genotype Frequency, *n*			
LL	3	1	2
ML	35	10	25
MM	149	73	76
MS	50	43	7
SL	2	0	2
SS	8	7	1
Allele Frequency			
L	0.09	0.04	0.14
M	0.78	0.74	0.81
S	0.14	0.21	0.05
CYP2C9*2 (rs1799853)			
Genotype Frequency, *n*			
CC	204	128	76
CT	42	6	36
TT	1	0	1
Allele Frequency			
C	0.91	0.98	0.83
T	0.09	0.02	0.17
CYP2C9*3 (rs1057910)			
Genotype Frequency, *n*			
A A	236	131	105
A C	11	3	8
Allele Frequency			
A	0.98	0.99	0.96
C	0.02	0.01	0.04
CYP2C8*8 (rs7900194)			
Genotype Frequency, *n*			
C C	239	126	113
C T	8	8	0
Allele Frequency			
C	0.98	0.97	1.00
T	0.02	0.03	0.00
rs12777823			
Genotype Frequency			
G G	153	68	85
G A	94	66	28
Allele Frequency			
G	0.81	0.75	0.88
A	0.19	0.25	0.12

**Table 4:** LD statistics between the tested alleles in the AA and EA samples.

	AA, D’(r^2^)			
	CYP2C9*2	CYP2C9*3	CYP2C9*8	rs12777823
pVNTR-S	0.19 (0.0002)	0.99 (0.05)	0.11 (0.001)	0.53 (0.22)
CYP2C9*2	1 (1)	0.78 (0.0002)	0.09 (0.007)	0.41(0.001)
CYP2C9*3		1(1)	0.83 (0.0002)	0.98 (0.004)
CYP2C9*8			1 (1)	0.99 (0.091)
	EA, D’(r^2^)			
	CYP2C9*2	CYP2C9*3	CYP2C9*8	rs12777823
pVNTR-S	0.99 (0.009)	0.99 (0.79)	N.D.	0.24 (0.02)
CYP2C9*2	1 (1)	0.98 (0.007)	N.D.	0.99 (0.02)
CYP2C9*3		1 (1)	N.D.	0.06 (0.001)
CYP2C9*8			N.D.	N.D.

N.D. not determined due to there being no CYP2C9*8 carriers in EAs.

**Table 5:** The association between CYP2C9 expression and the genotypes of pVNTR and the SNPs in the liver samples.

	AA + EA		AA Only		EA Only	
	Estimate ± SE (95% CI)	*p* Value	Estimate ± SE (95% CI)	*p* Value	Estimate ± SE (95% CI)	*p* Value
Without TFs						
pVNTR-S	−0.18 ± 0.08 (−0.35~−0.02)	0.032	−0.23 ± 0.10 (−0.44~−0.02)	0.028	0.11 ± 0.14 (−0.17~0.39)	0.450
CYP2C9*2	−0.14 ± 0.11 (−0.36~0.07)	0.185	0.08 ± 0.30 (−0.67~0.51)	0.782	−0.18 ± 0.09 (−0.36~0.01)	0.063
CYP2C9*3	−0.10 ± 0.19 (−0.49~0.28)	0.587	−0.68 ± 0.41 (−1.51~0.13)	0.103	0.04 ± 0.18 (−0.31~0.41)	0.799
CYP2C9*8	−0.02 ± 0.23 (−0.48~0.44)	0.931	−0.02 ± 0.26 (−0.49~0.55)	0.917	N.D.	N.D.
rs12777823	0.02 ± 0.08 (−0.15~0.19)	0.786	0.03 ± 0.12 (−0.20~0.28)	0.751	0.05 ± 0.11 (−0.15~0.27)	0.602
With TFs						
pVNTR-S	−0.18 ± 0.05 (−0.28~−0.07)	0.0007	−0.22 ± 0.07 (−0.36~−0.08)	0.002	0.02 ± 0.09 (−0.15~0.20)	0.805
CYP2C9*2	−0.03 ± 0.07 (−0.07~0.11)	0.677	−0.16 ± 0.20 (−0.56~0.24)	0.431	−0.02 ± 0.06 (−0.13~0.10)	0.762
CYP2C9*3	0.006 ± 0.12 (−0.24~0.25)	0.962	−0.02± 0.29 (−0.59~0.54)	0.923	−0.03 ± 0.11 (−0.26~0.19)	0.750
CYP2C9*8	−0.25 ± 0.15 (−0.55~0.05)	0.299	−0.25 ± 0.18 (−0.61~0.10)	0.169	N.D.	N.D.
rs12777823	−0.05 ± 0.05 (−0.17~0.05)	0.299	−0.10 ± 0.08 (−0.26~0.06)	0.234	0.04 ± 0.06 (−0.09~0.17)	0.536

N.D., not determined. NR1I3, PPARA, ESR1, FOXA2, AHR, and ARNT were included in the models with TFs. Estimate values are in Log_10_ scale.

## Data Availability

The original contributions presented in this study are included in this article/Supplementary Materials; further inquiries can be directed to the corresponding author.

## Supplementary Materials

The following supporting information can be downloaded at https://www.mdpi.com/article/10.3390/genes16020213/s1: Supplemental Figure S1: Interaction between pVNTR-S and ARNT in AA (a) and EA (b) cohorts.
