# Supplementary material for: The Association Between Promoter Tandem Repeat Polymorphism (pVNTR) and CYP2C9 Gene Expression in Human Liver Samples
Source: Genes (Basel). 2025 Feb 11;16(2):213. doi: 10.3390/genes16020213 (PMC11855013; doi:10.3390/genes16020213)
Supplement: Supplementary file 1 [file genes-16-00213-s001.zip › genes-3428865-supplementary.pdf]

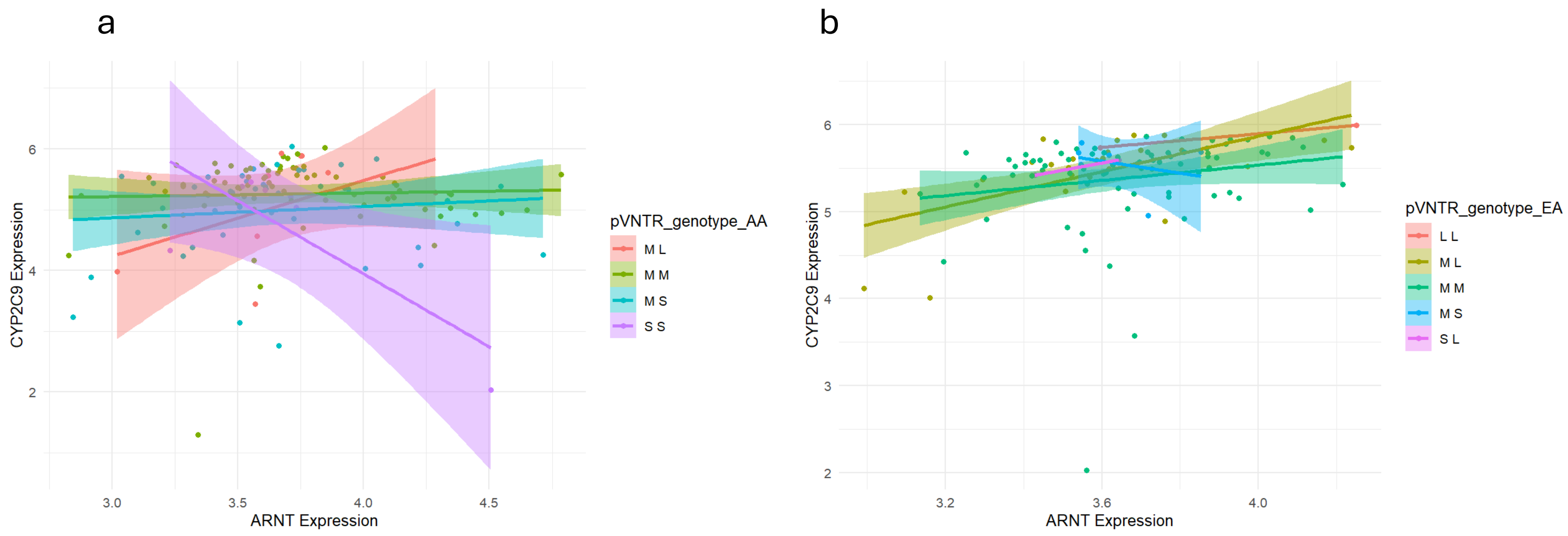

Supplemental Figure S1. Interaction between pVNTR-S and ARNT in AA (a) and EA (b) cohorts. Intersecting lines indicate an interaction, while parallel lines do not. The interaction was only significant in homozygous pVNTR-S samples in AA cohort.
